# Spectral-Based Screening Approach Evaluating Two Specific Maize Lines With Divergent Resistance to Invasion by Aflatoxigenic Fungi

**DOI:** 10.3389/fmicb.2019.03152

**Published:** 2020-01-22

**Authors:** Zuzana Hruska, Haibo Yao, Russell Kincaid, Feifei Tao, Robert L. Brown, Thomas E. Cleveland, Kanniah Rajasekaran, Deepak Bhatnagar

**Affiliations:** ^1^Geosystems Research Institute, Mississippi State University, MSU Science and Technology, Stennis Space Center, Starkville, MS, United States; ^2^Southern Regional Research Center, USDA-ARS, New Orleans, LA, United States

**Keywords:** fluorescence hyperspectral imaging, susceptible and resistant hybrids, aflatoxin screening, toxigenic and atoxigenic *Aspergillus flavus*, maize

## Abstract

In an effort to control aflatoxin contamination in food and/or feed grains, a segment of research has focused on host resistance to eliminate aflatoxin from susceptible crops, including maize. To this end, screening tools are key to identifying resistant maize genotypes. The traditional field screening techniques, the kernel screening laboratory assay (KSA), and analytical methods (e.g., ELISA) used for evaluating corn lines for resistance to fungal invasion, all ultimately require sample destruction. A technological advancement on the basic BGYF presumptive screening test, fluorescence hyperspectral imaging offers an option for non-destructive and rapid image-based screening. The present study aimed to differentiate fluorescence spectral signatures of representative resistant and susceptible corn hybrids infected by a toxigenic (SRRC-AF13) and an atoxigenic (SRRC-AF36) strain of *Aspergillus flavus*, at several time points (5, 7, 10, and 14 days), in order to evaluate fluorescence hyperspectral imaging as a viable technique for early, non-invasive aflatoxin screening in resistant and susceptible corn lines. The study utilized the KSA to promote fungal growth and aflatoxin production in corn kernels inoculated under laboratory conditions and to provide actual aflatoxin values to relate with the imaging data. Each time point consisted of 78 kernels divided into four groups (30-susceptible, 30-resistant, 9-susceptible control, and 9-resistant control), per inoculum. On specified days, kernels were removed from the incubator and dried at 60°C to terminate fungal growth. Dry kernels were imaged with a VNIR hyperspectral sensor (image spectral range of 400–1000 nm), under UV excitation centered at 365 nm. Following imaging, kernels were submitted for the chemical AflaTest assay (VICAM). Fluorescence emissions were compared for all samples over 14 days. Analysis of strain differences separating the fluorescence emission peaks of resistant from the susceptible strain indicated that the emission peaks of the resistant strain and the susceptible strains differed significantly (*p* < 0.01) from each other, and there was a significant difference in fluorescence intensity between the treated and control kernels of both strains. These results indicate a viable role of fluorescence hyperspectral imaging for non-invasive screening of maize lines with divergent resistance to invasion by aflatoxigenic fungi.

## Introduction

Aflatoxins are highly toxic and carcinogenic secondary metabolites predominantly produced by the *Aspergillus flavus* (*A. flavus*) and *A. parasiticus* fungi. When a susceptible crop (e.g., maize) is colonized by a toxigenic *Aspergillus* fungus, aflatoxins are produced and contaminate the grain and grain products, threatening human and animal health worldwide (Unnevehr and Grace, 2013). *A. flavus* is an opportunistic pathogen occurring with higher incidence on maize grown under stressed conditions preharvest, including late-season drought and high night temperatures during kernel filling and ear maturation, and under poor storage conditions post-harvest (Widstrom et al., 1987; Mahuku et al., 2013). Because of the ubiquitous nature of the *Aspergillus* fungus, aflatoxin contamination may occur at any point along the maize production line and in storage. Different pre- and post-harvest strategies for controlling aflatoxin in food and feed production have been explored and implemented over the years since its initial discovery in the 1960s; however, no permanent solution has yet been attained.

A noteworthy preventive strategy in the continued effort of mitigating aflatoxin contamination in food and/or feed grains focuses on developing host–plant resistance to eliminate aflatoxin from susceptible crops, including maize (Cary et al., 2011). In order to inhibit fungal colonization and subsequent toxin production, host resistance is a cost-effective approach, which preserves the environment in terms of harmful residue, and is compatible with other control measures, including biocontrol and appropriate storage practices (Mahuku et al., 2013). Natural resistance to *A. flavus* infection and subsequent aflatoxin production in maize was first discovered during the early 1980s (King and Scott, 1982; Gardner et al., 1987; Widstrom et al., 1987), with ongoing research adapting new technologies including next-generation sequencing and association mapping to identify gene sequences associated with aflatoxin resistance which would assist in developing aflatoxin-resistant varieties (Scott and Zummo, 1988; Campbell and White, 1995; Widstrom et al., 2003; Brown et al., 2013; Mahuku et al., 2013). The infection of maize kernels by *A. flavus* is subject to natural variability which may lead to inaccurate classification of resistant plants. Therefore, when isolating resistant germplasms, the selection of resistant genes depends on the even distribution of artificially induced fungal infection over a test field, and on the availability of high-throughput screening (Mahuku et al., 2013). To this end, screening tools are key to identifying resistant maize genotypes. In addition to the traditional field screening techniques, the kernel screening laboratory assay (KSA) has been an invaluable technique developed to study resistance to aflatoxin production in maize. The KSA measures seed-based (maize-host) genetic resistance in mature kernels and can effectively separate susceptible kernels from resistant maize (Brown et al., 1993, 1995). The assay is simple to perform in the laboratory, is independent of outdoor weather conditions, requires few kernels, and correlates favorably with field findings. Ultimately, the kernels must be crushed for aflatoxin determination and field trials are required for confirmation of resistance (Brown et al., 2013). Other screening methods used for evaluating corn lines for resistance to fungal invasion include analytical methods [e.g., enzyme-linked immunosorbent assay (ELISA) or high performance liquid chromatography (HPLC)] which require sample destruction, and more recently, non-invasive, optical-imaging and spectral-based techniques (e.g., near-infrared spectroscopy (NIR) and hyperspectral imaging (HSI)].

Near-infrared spectroscopy is based on absorption of electromagnetic radiation in the 780–2500 nm wavelength range (Nicolaï et al., 2007; Huang et al., 2008; Xia et al., 2019). The NIR spectra consist of broad wave bands from overlapping absorptions corresponding to combinations of C–H, O–H, and N–H bonds present in organic compounds, allowing for detection of biological material (Xia et al., 2019). Several studies reported the potential of NIR for rapid detection in different applications. In agricultural research, NIR was used for quality evaluation of farm products in various fruits and vegetables (Pasquini, 2018), and testing of seeds (Zhu et al., 2015). The seed research included testing seeds for constituents, such as starches, sugars, and proteins, for vigor, insect infestation, disease, seed viability (Zhu et al., 2015; Xia et al., 2019), and for variety discrimination of grass (Ren et al., 2008) and rice (Attaviroj et al., 2011) seed. NIR spectroscopy was also used for detecting kernel rot and mycotoxins in maize (Berardo et al., 2005) and it was suggested the methodology may be applicable as a screening tool in large-scale breeding programs for selecting genotypes resistant to fungal and fumonisin contamination (Lanubile et al., 2017).

Hyperspectral imaging systems integrate NIR spectroscopy with digital imaging, adding a spatial component to the spectral information provided by NIR spectroscopy, resulting in a three-dimensional “hypercube” dataset consisting of high spectral and high spatial information of a specific target (Xia et al., 2019). In the past decade, hyperspectral imaging expanded the potential of NIR technology and opened up new application opportunities for the innovative technology, particularly in agriculture (Pasquini, 2018). Allowing for simultaneous multi-kernel acquisition, hyperspectral imaging systems dramatically increased analysis of single kernels including wheat, cotton, and maize seeds. Near infrared HSI was used to detect insect damaged kernels in wheat (Singh et al., 2009), and to classify individual cotton seeds with respect to variety (Soares et al., 2016). Application of HSI systems for classification of maize seed varieties was reported in several recent studies. Feng et al. (2017) applied NIR–HSI with multivariate data analysis to discriminate between transgenic maize kernels. Zhang et al. (2012), Yang et al. (2015), and Wang et al. (2016) used HSI and chemometrics to discriminate different maize varieties.

Fluorescence hyperspectral imaging offers yet another option for non-invasive and rapid image-based screening. It is a technologically advanced take on the basic bright green-yellow fluorescence (BGYF) presumptive screening test originally introduced in the 1970s to the grain industry. The method employs a HSI system with an ultraviolet fluorescence excitation source. Fluorescence hyperspectral imaging has been extensively researched as a non-invasive tool in agriculture for assessing the quality and safety of food and feed in commodities exhibiting fluorescence properties (Kim et al., 2001; Chen et al., 2002; Carrasco et al., 2003; Kong et al., 2004; Ariana et al., 2006; Gowen et al., 2007; Zhang et al., 2012; Liu et al., 2015). In maize, the method was applied for detecting and classification of kernels contaminated with aflatoxins (Yao et al., 2006, 2010). Fluorescence imaging of maize plants was also utilized for detecting diseases in genetic disease resistance studies (Rascher et al., 2009).

We hypothesize that fluorescence hyperspectral imaging may be a viable technique for early, non-invasive aflatoxin screening of resistant and susceptible corn lines. The present study utilized the earlier mentioned KSA to promote fungal growth and aflatoxin production in corn kernels inoculated under laboratory conditions and to provide actual aflatoxin values, determined by chemical analysis, to relate with the image data. The specific objective of the study aimed to differentiate fluorescence spectral signatures of a representative resistant and a representative susceptible corn hybrid infected by a toxigenic and an atoxigenic strain of *A. flavus*, at several time points, in order to evaluate fluorescence hyperspectral imaging as a rapid and non-destructive screening technique of resistant and susceptible maize.

## Materials and Methods

### Maize Strains

**FIGURE 1:**
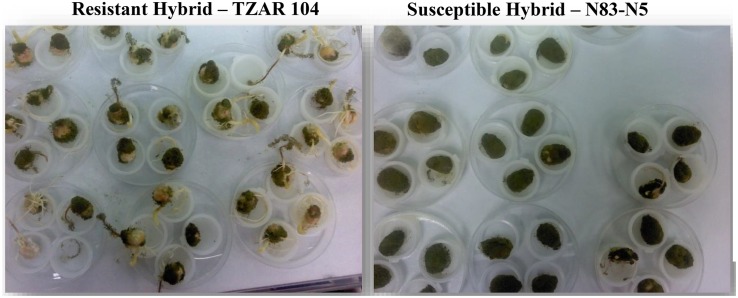
Examples of AF13 treated resistant and susceptible kernels showing differences in colonization. The susceptible hybrid exhibits a more robust fungal growth compared to the resistant hybrid.

To determine the viability of the use of fluorescence hyperspectral imaging to differentiate resistant and susceptible corn hybrids, one resistant and one susceptible corn line were utilized in this feasibility study. The resistant maize strain TZAR 104 obtained from FFS-SRRC-ARS-USDA, New Orleans, LA, United States, is a combination of African and southern US lines released in 2008. The N83-N5 (Liberty non-linked) hybrid seed, obtained from the LSU Ag Center, Baton Rouge, LA, United States, was chosen as the susceptible maize strain for its propensity to readily colonize maize kernels in the laboratory and in the field (Hruska et al., 2013). Typical differences of colonization between resistant and susceptible corn kernels inoculated with a toxin producing *A. flavus* are presented in Figure 1.

### Preparation of Inocula

A toxin producing (SRRC-AF13) and a non-toxin producing (SRRC-AF36) strains of *A. flavus* were cultured on potato dextrose agar (PDA) media at 30°C in the dark. Several replicates of each fungus were cultured on separate plates. Conidia were harvested on 5th day of growth and suspended in buffer at a dilution of 4 × 10^6^ conidia/mL, determined with a hematocytometer. The inocula were stored in separate containers at 4°C.

### Sample Preparation – *In situ* Inoculation

The resistant and the susceptible maize kernels were inoculated with both strains of *A. flavus* (AF13 and AF36) and placed with their respective controls, into an incubator at 31°C. The kernels were surface sterilized in 70% ethanol (4 min) followed by three washes (1 min each) in distilled water before being placed into an inoculum made from each *A. flavus* culture at a standard dilution of 4 × 10^6^ spores/mL and stirred for 1 min. The experiment was conducted in two parts. The first part involved only the toxigenic fungus and the second part involved the atoxigenic fungus. Each part of the experiment consisted of four trays per treatment day (susceptible, resistant, susceptible control, and resistant control). Each treatment tray contained 10 dishes with 3 kernels/dish, 30 kernels total. Each control tray contained three small plates with three kernels/plate, nine kernels total. Treatment was terminated on Days 5, 7, 10, and 14. On the specified days, the four trays designated for that day (78 kernels/treatment) were removed from the incubator, each plate from all trays was transferred into pre-labeled coin envelopes (one bag/dish/three kernels) and placed into a 60°C oven for 2 days to terminate fungal growth.

### Imaging Procedure

Dry kernels were removed from the oven, wiped off from excess mold, and placed on a pre-labeled ceramic tray identifying each individual kernel with a grid. The tray contained 30 shallow indentations, each to accommodate a single corn kernel. It was painted with a flat, black paint to reduce reflection and to enhance the contrast between the background and the samples. The kernels were imaged using the VNIR hyperspectral sensor (VNIR 100E, MSU, Stennis Space Center, MS, United States) with image spectral range of 400–1000 nm, under UV (Model XX-15A; Thermo Fisher Scientific Inc., Waltham, MA, United States) excitation centered at 365 nm (Yao et al., 2010). A dark current calibration image was also taken. Following imaging, kernels were returned into the specified coin envelopes and chemically analyzed using the AflaTest assay (VICAM).

### Image Analysis

The fluorescence hyperspectral images were preprocessed where the sensor background noise was removed through dark current subtraction, image band wavelengths were assigned, and random noise was removed via Savitzky–Golay filtering. Each preprocessed image had a range from 400 to 700 nm. The corn fluorescence spectra were extracted from individual corn kernels and averaged for each three-kernel sample. In order to detect differences in the spectral pattern of toxin producing and non-toxin producing *A. flavus* on susceptible and resistant kernels over a 14-day growth period, mean fluorescence spectra were obtained through spatial subset of each image (region of interest) along with standard deviations. Fluorescence emissions were compared for all three-kernel samples over 14 days. Spectral features including peak locations (wavelengths which correspond with the maximum fluorescence intensity values) and average fluorescence intensities were extracted and used in statistical analyses.

### Chemical Analysis

Imaged corn samples were chemically analyzed for aflatoxin content using the AflaTest from VICAM (VICAM, Milford, MA, United States). The AflaTest is an approved method by the United States Department of Agriculture Federal Grain Inspection Service, for quantitative analysis of aflatoxin in maize. Details of the method were described previously (Hruska et al., 2013). Briefly, the three-kernel samples were weighed, crushed, and extracted with methanol/water (80/20%). Extracts were diluted, filtered, and passed through AflaTest columns. The columns were washed with distilled water and eluted with 100% methanol. Eluted samples were mixed with developer (1:1) and aflatoxin (ppb) was measured in the EX-4 series Fluorometer (VICAM).

### Statistical Analysis

Two sets of image data based on side (germ or embryo side, and the opposite endosperm side) were analyzed separately, using a 2 × 2 factorial design (resistant, susceptible) and (toxigenic, atoxigenic). Spectral emission peak locations were extracted from the preprocessed fluorescence data and group differences between spectral peaks over a 14-day incubation period were analyzed using analysis of variance (ANOVA) in MATLAB followed by *post hoc t*-test analyses. Results were plotted in Microsoft Excel 360. Results of the chemical analysis were also analyzed with an ANOVA to determine group differences, in MATLAB and plotted in Microsoft Excel.

## Results

**FIGURE 2:**
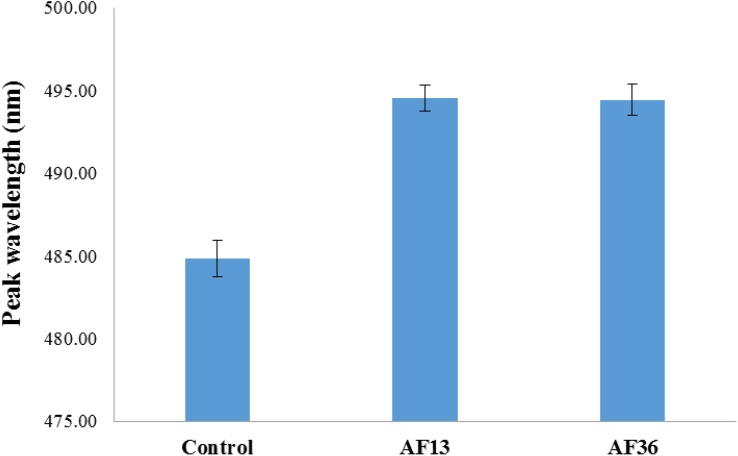
Bar graph representation of entire treatment image data set including standard error bars. Fluorescence emission peaks of both AF13 and AF36 inoculated kernels were significantly different from the uninoculated controls (*p* < 0.001), but not from each other.

The treatment analysis results of the entire image data set, irrespective of side (germ, endosperm), are presented in Figure 2. A main effect of treatment (*p* < 0.001) with *post hoc* analysis shows that the fluorescence emission peaks of both AF13 and AF36 inoculated kernels were significantly different from the untreated controls (*p* < 0.001), but not from each other.

**FIGURE 3:**
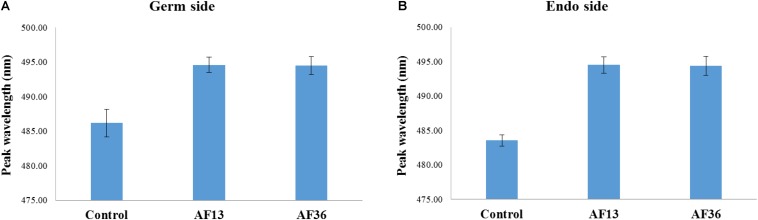
Bar graph representation of germ **(A)** and endosperm **(B)** image data sets, with respect to treatment. Fluorescence emission peaks of both AF13 and AF36 inoculated kernels were significantly different from the controls (*p* < 0.001), but not from each other.

Similar results were revealed when each side of kernels was analyzed separately. A main effect of treatment (*p* < 0.001) with *post hoc* analysis shows that in both treatment groups (AF13 and AF36) the emission peaks can be differentiated from the peaks of the controls, but not from each other on both the germ and the endosperm sides of imaged kernels (Figures 3A,B). Therefore, the data were pooled and no further distinction, with respect to side, was made in the subsequent analyses.

**FIGURE 4:**
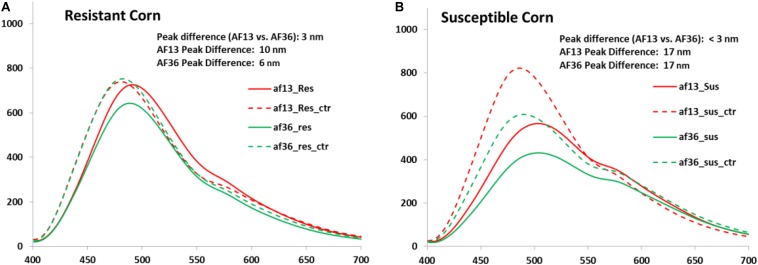
Mean spectra of **(A)** resistant kernels and **(B)** susceptible kernels inoculated with AF13 and AF36 with their respective controls. A significant fluorescence peak shift is evident in both the resistant and susceptible kernels between the inoculated kernels and the controls.

Effects of the inoculation treatment on the resistant and susceptible kernels are illustrated by the image data (Figure 4). In the resistant corn group, the mean peak difference between the AF13 inoculated and the AF36 inoculated kernel emissions was 3 nm, while the mean peak difference between the AF13 inoculated and AF36 inoculated kernel and their respective control emissions was 10 and 6 nm (Figure 4A). The mean peak difference between AF13 inoculated and AF36 inoculated kernel emissions was <3 nm, while the mean peak difference between both inoculated groups and their respective controls was 17 nm in the susceptible kernels (Figure 4B).

**FIGURE 5:**
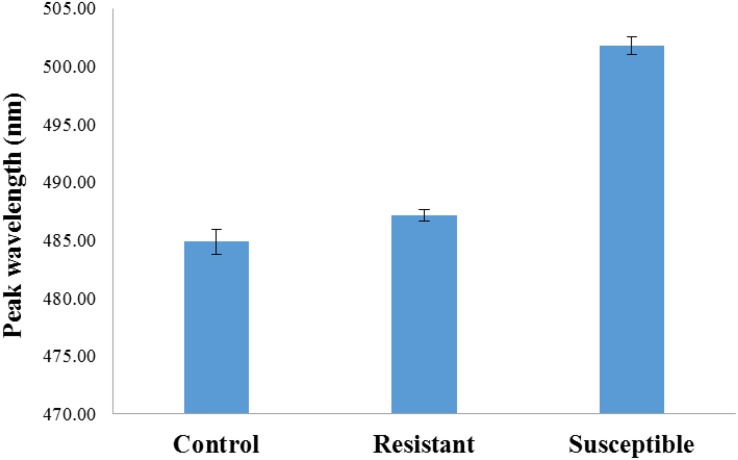
Bar graph representation of strain differences (susceptible and resistant). There was a main effect of strain *p* < 0.01 separating the two inoculated strains from each other. The difference between the uninoculated controls and the inoculated resistant kernels was not significant.

Statistical analysis of strain differences separating the fluorescence emission peaks of resistant and the susceptible strain (Figure 5) indicates a main effect of strain (*p* < 0.01) where the emission peaks of the resistant strain and the susceptible strain are significantly different from each other. Although emission peaks from both corn varieties inoculated with both fungi differed from the uninoculated controls, the difference between the controls and the resistant kernels was not significant.

**FIGURE 6:**
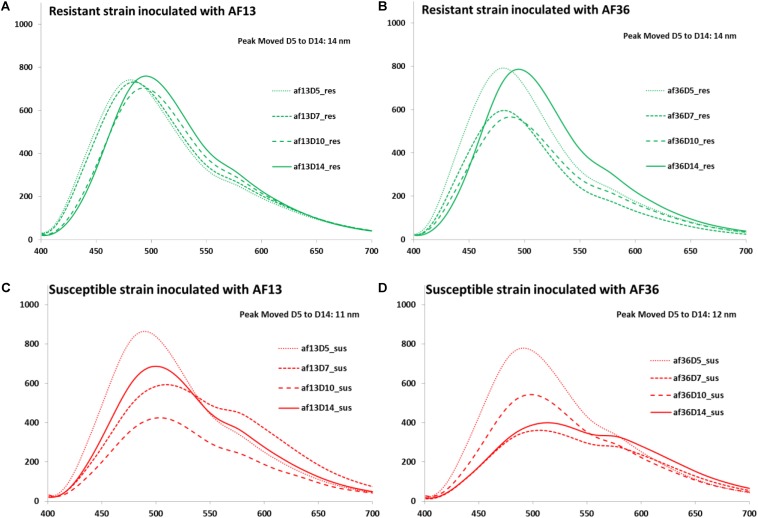
Mean spectral curves of image data over the 14-day incubation period: **(A)** AF13 inoculated resistant corn strain **(B)** AF36 inoculated resistant corn strain **(C)** AF13 inoculated susceptible corn strain and **(D)** AF36 inoculated susceptible corn strain. A fluorescence peak shift toward longer wavelengths was observed in the spectra from both corn varieties inoculated with each fungal strain from Days 5 to 14 post-inoculation. In addition to the shift in the peak location, there was a marked difference in the general appearance of the spectral curves between the AF13 and the AF36 inoculated kernels on different days post-inoculation particularly in terms of the intensity of the fluorescence.

The next step in the analysis involved the growth of the fungus and aflatoxin accumulation at four different time points post inoculation (Days 5, 7, 10, and 14). Mean spectral curves of image data over the 14-day incubation period are presented in Figure 6. An expected fluorescence peak shift toward longer wavelengths was observed in the spectra from both corn varieties inoculated with each fungal strain from Days 5 to 14 post-inoculation. In the resistant corn, both the AF13 (Figure 6A) and the AF36 (Figure 6B) inoculated kernels exhibited a 14-nm fluorescence peak shift over the incubation period. In the susceptible corn, the peak shifted 11 nm in the AF13 (Figure 6C) inoculated samples and 12 nm in the AF36 (Figure 6D) inoculated corn over the same 14-day period.

**FIGURE 7:**
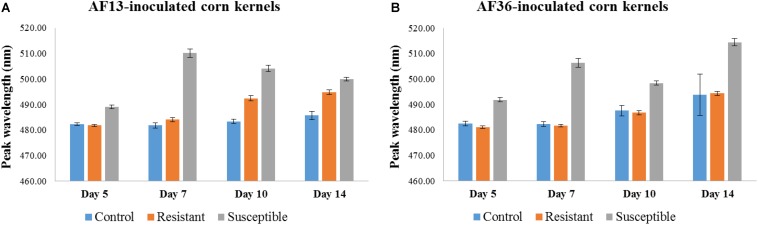
Bar graph representation of mean peak locations of control, resistant, and susceptible corn kernels inoculated with **(A)** AF13 and **(B)** AF36 fungal strains. A significant peak shift was apparent in the AF13-inoculated kernels, differentiating the susceptible group from the resistant and control groups on Days 5 and 7. By Day 10 peak locations of all three groups were significantly different from each other *p* < 0.01. In the AF36-inoculated kernels, the peaks of the susceptible group were significantly different from the other two groups *p* < 0.01; however, the peaks of the resistant and control groups were not significantly different from each other at any of the four time points.

Statistical analysis of AF13-inoculated kernels (Figure 7A) revealed that peak locations of the susceptible kernels were significantly different from peak locations of both the control and resistant kernels on Days 5 and 7, *p* < 0.01; however, the difference between the peaks of the control and the resistant kernels was not significant. By Day 10, persisting through Day 14, the peak location of each group was significantly different from the other two groups, *p* < 0.01. Analysis of the AF36-inoculated kernels (Figure 7B) found that although the peak location of the resistant and susceptible groups differed early on (Day 5), *p* < 0.01, the peaks of the resistant group did not differ from those of the control group of kernels through the 14-day incubation period.

**FIGURE 8:**
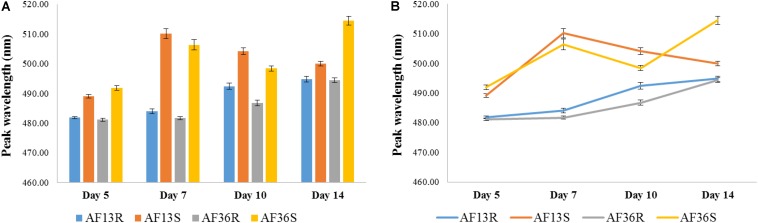
Bar **(A)** and point **(B)** graph representation of mean peak locations of the four major groups (AF13-resistant AF13R; AF13-susceptible AF13S; AF36-resistant AF36R; AF36-susceptible AF36S) at each time point post-inoculation. An overall main effect of Day was found with *p* < 0.01. A significant difference between the peak location means of all four treatment groups was found only on Day 10. The point graph **(B)** revealed a time-dependent interaction in the two susceptible groups, where the wavelength of the peak mean of the AF36S group gradually increased with time, while that of the AF13S increased to Day 7 and then decreased through Day 14.

Figure 8 summarizes the analyses of the four major groups (AF13-resistant AF13R; AF13-susceptible AF13S; AF36-resistant AF36R; AF36-susceptible AF36S) at each time point post-inoculation in terms of mean peak locations. An overall main “Day” effect was found with *p* < 0.01. On Day 5, peak locations of both resistant groups, AF13R and AF36R, were significantly different from both susceptible groups, AF13S and AF36S, *p* < 0.01. And the mean peak location of each susceptible group, AF13S and AF36S, was significantly different from all the other three groups, *p* < 0.01. On Day 7, peak locations of both resistant groups, AF13R and AF36R, were significantly different from both susceptible groups, AF13S and AF36S, *p* < 0.001. However, there was no significant difference between the mean peak location of the two resistant or the two susceptible groups on Day 7 post-inoculation. Interestingly, by Day 10, a significant difference was found between the peak location means of all four treatment groups. Fourteen days post-inoculation, statistical analysis found similar differences seen previously on Day 5. The peak means of both resistant groups, AF13R and AF36R, were significantly different from both susceptible groups, AF13S and AF36S, *p* < 0.01. And the peak location of each susceptible group, AF13S and AF36S, was significantly different from all the other three groups, *p* < 0.01. The point graph representation of the data (Figure 8B) revealed a time-dependent interaction most apparent in the two susceptible groups, where the wavelength of the peak mean of the AF36S group gradually increased with time, while that of the AF13S increased to Day 7 and then decreased through Day 14.

**FIGURE 9:**
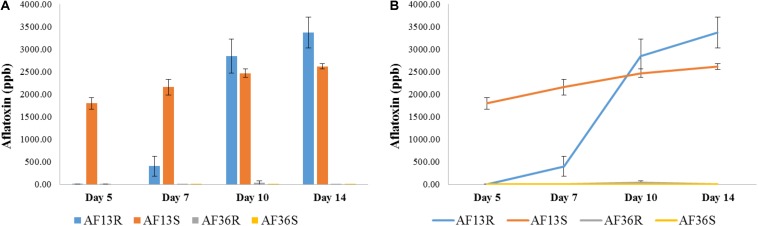
Bar **(A)** and point **(B)** graph representing results of the AflaTest chemical analysis. An overall main effect of strain (resistant, susceptible) was revealed with *p* < 0.001. A time-dependent interaction between the AF13R and AF13S groups was found **(B)** where the aflatoxin concentration of AF13S started high and increase gradually with time, while that of the AF13R had a slow start through Day 7 and then increased exponentially through Day 14.

The results of the AflaTest chemical analysis are presented in Figure 9. The two groups of kernels inoculated with the atoxigenic AF36 fungal strain (AF36R and AF36S) were not expected to test positive for aflatoxin; therefore, positive aflatoxin reading in any of those kernels was attributed to internal contamination naturally acquired in the field. Statistical analysis involved mainly the two AF13-inoculated groups of kernels, AF13R and AF13S, with the other two groups serving as controls and referred to as such, only in this section. An overall main effect of strain (resistant, susceptible) was revealed with *p* < 0.001 (Figure 9A). On Day 5, the aflatoxin level of AF13S was already significantly different from AF13R, which at this point did not differ from the control (AF36) groups. Similar results were noted on Day 7. Although the AF13R group showed some aflatoxin contamination, it was not significantly different from the control groups. By Day 10, aflatoxin levels in the AF13R group surpassed the levels found in the AF13S group; however, the difference was not significantly different until Day 14 (*p* < 0.01). The point graph (Figure 9B) revealed a time-dependent interaction between the AF13R and AF13S groups, where the aflatoxin concentration of AF13S started high and increase gradually with time, while that of the AF13R had a slow start through Day 7 and then increased exponentially through Day 14, significantly overtaking the aflatoxin concentration of the susceptible group.

## Discussion

Aflatoxins in food and/or feed pose acute and chronic risks to health of people and animals. In human populations, consumption of high levels of aflatoxins can result in acute illness or even death (Strosnider et al., 2006). Although aflatoxin contamination is a global problem, the areas most affected are poverty stricken tropical countries south of the Sahara in Africa, and Southern Asia where maize and groundnuts are the diet staples most often contaminated with aflatoxins. In addition to acute effects, chronic exposure to aflatoxins were found to be associated with liver cancer (Wu, 2013), childhood growth stunting (Gong et al., 2002, 2004; Khlangwiset et al., 2011), and immune suppression (Williams et al., 2004; Jiang et al., 2005). The global burden of liver cancer attributed to aflatoxin exposure was estimated to be approximately 23% of all liver cancer cases per year (Liu and Wu, 2010; Liu et al., 2012). This is significant, considering most of the people will die within 3 months of diagnosis. In animal studies, adverse effects were also found on health, growth, and productivity when animals were fed feed contaminated with mycotoxins.

General aflatoxin exposure can be reduced by improved field, harvesting, and storage practices, and by switching to crop hybrids less prone to aflatoxin contamination. Together with pre- and post-harvest strategies, and effective screening tools, host plant resistance is considered to be a practical and effective approach in reducing aflatoxin contamination in maize (Brown et al., 2016).

In this study we took the opportunity to evaluate fluorescence HSI as a viable technique for early, non-invasive aflatoxin screening of resistant and susceptible maize hybrids infected by a toxigenic and an atoxigenic strain of *A. flavus*, for potential in-field domestic as well as international applications.

Hyperspectral imaging successfully differentiated the resistant from the susceptible maize kernels based on the location of the fluorescence emission peaks regardless of the treatment received. The resistant kernels could not be differentiated from the uninoculated controls when all data were collapsed across treatments and duration of the study. Similarly, both treatment groups were differentiated from the untreated controls, when the data were collapsed across strain and time, but the treatments could not be separated from each other. However, data analysis with respect to days revealed interesting insights of the general trends which were obscured when the data were analyzed as a whole.

In terms of the maize variety data, in the AF13-inoculated kernels, both the resistant and the susceptible kernels could be separated from the controls and from each other by day 10. Although the susceptible kernels were separable from the resistant and the control kernels as early as Day 5, the resistant kernels were not separable from the controls until Day 10. In the AF36 inoculated maize, the susceptible kernels were separable from the resistant and the control kernels on Day 5, but the resistant kernels were not separable from the controls over the duration of the experiment. These results revealed a temporal window where it was possible to differentiate AF13 inoculated resistant kernels from the AF36 inoculated resistant kernels, which was Day 10 post-inoculation. Of course, these results are specific to the two strains (resistant and susceptible) used in the current experiment, and additional strains must be tested before a more generalized conclusion may be inferred. Interestingly, Day 10 was also significant when the four groups (two resistant and two susceptible) were analyzed on different days post-inoculation. Day 10 was the only day where all four groups differed from each other.

Aflatoxin-resistant variety characteristics may include direct resistance to fungus and aflatoxin accumulation, indirect resistance or tolerance to biotic and abiotic stresses, or morphological traits such as ear, kernel, and husk characteristics that impede or delay fungal access or growth. Sources of resistance to many of these factors have been identified and are now being combined to develop aflatoxin-resistant maize germplasm adapted to various agricultural ecosystems (Brown et al., 2013; Mahuku et al., 2013). The resistant TZAR104 maize variety used in the current study is one of the six maize germplasm lines released by the International Institute of Tropical Agriculture-Southern Regional Research Center (IITA-SRRC) maize breeding collaboration for use in African National Programs and U.S. maize breeding programs (Menkir et al., 2006). TZAR104 was extracted from a backcross involving GT-MAS:gk (U.S. inbred line, with proven resistance to aflatoxin contamination) as a recurrent parent and KU1414-SR (tropical elite African inbred line with some level of resistance to aflatoxin production) as a non-recurrent parent (Brown et al., 2016). Therefore, the resistance exhibited by the TZAR104 kernels is a combination of resistant traits from both the U.S. and African lines to aflatoxin production and contamination based on direct resistance to diseases such as *Aspergillus* ear rot (Brown et al., 2001). In the present experiment, it appears that the resistant maize was resistant to the AF36 infection over the course of the study (Figure 7B). The resistant strain also resisted the AF13 infection, where it took longer for the infection to occur compared to the susceptible strain, but by Day 10 the resistant kernels were infected, as revealed by the spectral data (Figure 7A). The chemical data also supported these findings. The aflatoxin contamination levels in the resistant kernels reached those of the susceptible kernels by Day 10 and surpassed them by Day 14 (Figures 9A,B). The enhanced resistance may be partially attributed to the rounder shape and flinty texture of the TZAR104 kernels (Brown et al., 2016) which may have been harder to penetrate than the usual dent corn seed coat. It appears that the resistance is limited to penetrating the pericarp (Guo et al., 1995), and once the barrier is breached the infection spreads rapidly.

Fluorescence intensities in the image data were also examined in the different treatment groups for each variety. There was a noticeable difference in intensities between the resistant and the susceptible corn. In the resistant corn, the difference in intensities between the AF13 and the AF36 treated groups compared to their respective controls was insignificant. Although there was a slight difference in fluorescence intensity between the AF13 treated and the AF36 treated groups, with the intensity of the AF36 group being lower than that of the AF13 treated group, this too was not significant (Figure 4A). In the susceptible corn, however, this difference was much greater, where fluorescence intensities of the treatment groups were between ∼25 and 30% lower than their respective controls (Figure 4B). Additionally, the intensity of the AF13 treated group was ∼20–25% higher than that of the AF36 treated group. This agrees with a previous study which examined internal fluorescence emissions associated with aflatoxin contamination from corn kernel cross-sections inoculated with toxigenic and atoxigenic *A. flavus*. The study found that on Day 9 post-inoculation, it was not possible to separate the AF13 inoculated kernels from the uninoculated controls based on fluorescence peak locations. However, they noticed that the intensity of the AF13-induced fluorescence in the endosperm was approximately double that of AF36 (Hruska et al., 2017).

Over the course of the study, the two maize strains were easily distinguished from the early days of the experiment, where fluorescence signals from the susceptible corn line were different from the resistant line on Day 5 in terms of the peak location and on Day 7 in terms of both the peak location and the fluorescence intensity. It was also possible to differentiate the inoculated resistant line from the uninoculated controls from the seventh day post-inoculation based on fluorescence intensity, which was not possible until day 10, in the current study, when taking only the change in peak location into consideration. This indicates that fluorescence intensity has a relevant, or even a crucial role in HSI-based aflatoxin detection, along with the difference in peak location, when evaluating contaminated and uncontaminated maize from diverse susceptible and resistant lines. Follow up fluorescence HSI studies need to evaluate additional resistant and susceptible maize varieties focusing on the fluorescence emission intensities along with the fluorescence peak shifts to estimate the earliest time point of detecting aflatoxin contamination in maize in order to protect food and feed streams from potential adverse effects on global health.

## Conclusion

Using non-invasive HSI technology, the present *in situ* study found that the resistant strain, TZAR 104 was resistant to the atoxigenic AF36 fungal inoculum over the 14 days of the study, while it only temporarily resisted the entry of the toxin-producing AF13 fungus, based on spectral data. The resistance appeared limited to penetrating the seed-coat, because once the barrier was breached the infection and associated toxin accumulation spread rapidly. The study also revealed a significant role for the intensity of fluorescence when using fluorescence hyperspectral imaging for early detection of maize kernels infected with toxigenic and atoxigenic *A. flavus* in resistant and susceptible corn varieties. While the study confirmed the usefulness of fluorescence HSI as a rapid and non-destructive tool for screening different varieties of maize infected with aflatoxins, it is important that follow-up studies focus on the relationship between the fluorescence peak shifts and the fluorescence intensities to determine the optimum timing indicators for the earliest detection of aflatoxins in maize, with hyperspectral fluorescence.

## Data Availability Statement

The datasets generated for this study are available on request to the corresponding author.

## Author Contributions

All authors participated in planning the experiments and edited the manuscript. ZH designed and executed the experiments, interpreted the data, and wrote the manuscript. HY and RB oversaw the implementation of the experiments. RK collected and processed the spectral images. FT performed the statistical analysis of the image and aflatoxin data.

## Conflict of Interest

The authors declare that the research was conducted in the absence of any commercial or financial relationships that could be construed as a potential conflict of interest.
